# Mr. Foster, on an Extraordinary Case of Monstrosity

**Published:** 1799-10

**Authors:** Thomas Foster

**Affiliations:** Surgeon


### *To the Editors of the Medical and Phyſical Journal.*

GENTLEMEN,

I LAST Saturday delivered a woman of this place, who had gone her full time, of a male child; both hands, one knee, one leg, and one foot of which were either ſingularly imperfect, or ſtrangely diſtorted, according to the following deſcription. It lived ſome hours, and was perfect in every other part. The parents would neither ſuffer a drawing of it to be taken while it was alive, nor the body to be diſſected after it was dead. Being a ſtranger to you, I have taken care to have the fact atteſted by two gentlemen, who likewiſe ſaw and examined the infant.

I embrace this opportunity of wiſhing ſucceſs to your very valuable Publication, and am, with great reſpect,

Gentlemen, your moſt obedient ſervant,

THOMAS FOSTER, *Surgeon.*

*The Right Hand*—Was furniſhed with a thumb and fore-finger, perfect in their nails and joints; the other three were wanting. The ſpace they ſhould have occupied had much the appearance of a ſtump that had been healed after amputation. From the inner part of the wriſt were ſuſpended, by a ſlender filament, about an inch in length, two round fleſhy ſubſtances, one of them as large as a marble, the other ſomewhat ſmaller.

*The Left Hand*—Was deſtitute of both wriſt and palm, and terminated in one large finger, which had its nail and joints perfect, and was ſupported by a ſingle metacarpal bone, that moved upon the ſmall extremity of the ulna.

*The Left Knee*—Was deſtitute of a patella. A preternatural elongation of the thigh-bone impeded the outward motion of the leg, which was bent conſiderably inwards, and could move only backwards, and a little towards either ſide.

*The Left Leg*—Terminated abruptly in the baſis of the tibia, as if the foot had been long amputated from it.

*The Left Foot*—Was joined to the lower part of the leg in a horizontal direction. It had the fourth and fifth toes only. The ſpace which the others ſhould have occupied reſembled the defective part of the right hand. A kind of corn, evidently the effect of preſſure, grew from the outward ancle.

"I examined the child that my friend has above described, an hour or two after its birth. It was then alive, but not likely to live, and the foregoing description of it appears to me to be perfectly accurate.

"EDWARD FOSTER,"

HAMPTON-LUCY, *Aug.* 19, 1799. *Assist. Surg.* 64*th Reg. of Foot.*

"I baptized the child which my neighbour, Mr. FOSTER has here described, about a quarter of an hour after it was born. It died a few hours afterwards. The description appears as correct as possible.

"JOHN MORLEY."

*Vicar of Wasperton, and Curate of Hampton Lucy.*

